# Recent Trends in Chemical Sensors for Detecting Toxic Materials

**DOI:** 10.3390/s24020431

**Published:** 2024-01-10

**Authors:** Yeonhong Kim, Yangwon Jeon, Minyoung Na, Soon-Jin Hwang, Youngdae Yoon

**Affiliations:** Department of Environmental Health Science, Konkuk University, Seoul 05029, Republic of Korea; k99yh@konkuk.ac.kr (Y.K.); ywjun69@konkuk.ac.kr (Y.J.); nmy0611@konkuk.ac.kr (M.N.); sjhwang@konkuk.ac.kr (S.-J.H.)

**Keywords:** chemical optical sensor, electrochemical sensor, nanomaterial, biosensor, transcription factor, toxic material

## Abstract

Industrial development has led to the widespread production of toxic materials, including carcinogenic, mutagenic, and toxic chemicals. Even with strict management and control measures, such materials still pose threats to human health. Therefore, convenient chemical sensors are required for toxic chemical monitoring, such as optical, electrochemical, nanomaterial-based, and biological-system-based sensors. Many existing and new chemical sensors have been developed, as well as new methods based on novel technologies for detecting toxic materials. The emergence of material sciences and advanced technologies for fabrication and signal-transducing processes has led to substantial improvements in the sensing elements for target recognition and signal-transducing elements for reporting interactions between targets and sensing elements. Many excellent reviews have effectively summarized the general principles and applications of different types of chemical sensors. Therefore, this review focuses on chemical sensor advancements in terms of the sensing and signal-transducing elements, as well as more recent achievements in chemical sensors for toxic material detection. We also discuss recent trends in biosensors for the detection of toxic materials.

## 1. Introduction

Anthropogenic activities, including industrial development and manufacturing, are responsible for various types of environmental pollution caused by the release of toxic chemicals [1]. These chemicals include airborne toxic chemicals, medicines, heavy metals, and the byproducts of anthropogenic activities. Although often generated for the good of humankind, failure to effectively manage or control such materials threatens both the environment and human health [2]. The release of harmful chemicals (such as phenols, polycyclic aromatic hydrocarbons, herbicides, insecticides, and nitroxides) into the environment can disrupt the balance between ecosystems and human well-being [3,4]. Therefore, tools and methods for detecting and monitoring toxic materials are crucial for protecting human health. As we live in an era of abundant material development, it is also important to characterize and assess the risks of newly developed materials. Many countries, including South Korea, have implemented laws such as “The Act on Registration and Evaluation of Chemicals” to document the chemical and physical properties of these materials [5]. Although these systems are strictly controlled by national authorities, toxic materials must also be thoroughly monitored to avoid potential threats to human health.

Traditionally, environmental monitoring of toxic chemicals has been performed using instrument-based analyses, such as spectrophotometry, high-performance liquid chromatography, gas chromatography, and mass spectrometry [6,7,8]. However, despite the superior sensitivity and precision of these tools, demand has increased for other methods that can compensate for the disadvantages of instrument-based analyses, such as high instrument and time costs. Chemical sensors represent a rapid and simple alternative method of target detection. As such, multiple types of chemical sensors with various applications have been developed using the novel techniques of different research fields.

Chemical sensors transform chemical information, ranging from the concentration of a specific sample component to the total composition, into an analytically useful signals [9]. In other words, chemical sensors systems can recognize and report signals originating from the chemical reaction of an analyte or changes in its chemical and physical properties. The IUPAC Commission defines chemical sensors as follows: “analytical chemical sensors are miniaturized transducers that selectively and reversibly respond to chemical compounds or ions and yield electrical signals which depend on the concentration” [10]. Janata et al. documented the definitions and types of existing chemical sensors [11]. Chemical sensing involves a data collection process that identifies the chemical components in a system by measuring the chemical and physical property changes induced by the interaction between targets and sensing platforms. Based on this unique interaction, diverse data acquisition techniques have been applied to develop many types of chemical sensor, including thermal, electrochemical, potentiometric, and optical techniques [12,13,14]. Although traditionally classified according to the method of data transduction, such as electrochemical responses, optical responses, electrical signals, optical signals, changes in electrical properties, and mass changes, chemical sensors can also be classified according to the materials used for the sensing devices; for example, nanostructure-based sensors, carbon nanotube-based sensors, biological system-based sensors, and graphene-based sensors [15,16,17,18,19]. Since the chemical sensors were designed using a combination of sensing and signal transducing elements, the types of chemical sensors could be diversified enormously. In this regard, it is not meaningful to classify chemical sensors simply by their signal transducing/translating type or by their sensing elements.

Many existing review articles have effectively summarized the principles and mechanisms of various types of existing chemical sensors, including nanostructured material-based sensors, carbon nanotube sensors, graphene-based sensors, nanomaterial-based sensors, and biological system-based sensors, which are also categorized as electrochemical and optical chemical sensors [12,15,20,21,22,23]. Biosensors that use biomolecules such as enzymes, proteins, and living cells as composites for sensing and transducing signals represent a subclass of chemical sensors [10,24]. As a review of all chemical sensors would be impractical because of their substantial diversity and the continual development of new biosensor techniques, this review focuses on recent trends in chemical sensors used to detect toxic chemicals and discusses recent achievements in novel chemical sensor technologies. First, we discuss the applications and prospects of chemical sensors and introduce different types of chemical sensors for detecting toxic chemicals. We then discuss recent findings and applications of chemical sensors for toxic chemical monitoring, including transcription factor (TF)-based biosensors.

## 2. Overview of Chemical Sensors for Detecting Toxic Materials

**Figure 1:**
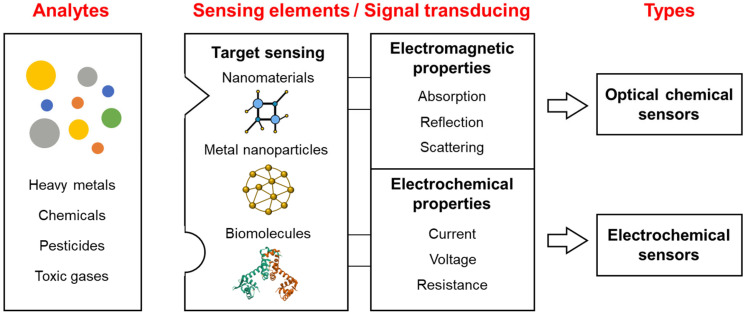
Types of chemical sensors based on sensing and transducing elements.

Chemical sensors are divided into various subclasses based on the materials used for the sensing elements and the types of output signal. Nonetheless, the basic principles and working mechanisms of chemical sensors are similar in terms of the major components, i.e., sensing elements and signal-transducing elements. The basic components of chemical sensors are shown in Figure 1. Chemical sensors comprise target sensing and signal-transducing elements and vary according to the sensing element source and type of signal-transducing element. Output type is also a significant factor in the classification of chemical sensors. Although chemical sensors are classically divided into optical, electrochemical, and thermal sensors based on the type of output signal, classification based on the sensing element has become more complicated. Chemical sensors that employ biomolecules as components are now categorized as biosensors. Owing to advances in materials science and fabrication technologies, new types of sensing element have recently been developed. Several types of chemical sensors detect the same target, and some chemical sensors can detect several targets via the sensing elements. For example, Balaji et al. reported that an optical sensor employing nanostructured cages as the sensing element can detect various metal ions including Sb, Hg, Pb, and Cd by modifying the cage structures [25]. In addition, methyl parathion, a component of pesticides, can be detected using electrochemical sensors that employ nanostructure-based sensing elements [26,27]. Therefore, the performance of chemical sensors can be determined using the target selectivity of the sensing elements and the sensitivity of the signal-transducing elements. From this perspective, new chemical sensors can be developed by combining sensing and signal-transducing elements.

The purpose of chemical sensors is to detect targets, including gases, chemicals, biomolecules, and cells, for diagnosis, monitoring, or determining production efficiency. However, considering the abundance of toxic materials released into the environment, the major application of chemical sensors is the detection of harmful materials that adversely affect human health. Among the many types of chemical sensors reported in different research fields, we focus on three types: optical, electrochemical, and biological system-based sensors.

## 3. Optical Chemical Sensors

Optical sensors are a group of chemical sensors that employ electromagnetic radiation as the energy source to induce transduction signals in the presence of targets. As described above, optical chemical sensors comprise target sensing (recognition) and signal-transducing elements; however, signal transduction is based on various optical principles, such as absorbance, emission, reflectance, and fluorescence. Therefore, direct spectroscopic methods such as UV spectroscopy and Raman spectroscopy are also considered optical sensors. In this review, according to the definition of chemical sensors, we include only sensors that employ changes in electromagnetic radiation as signal-transducing elements. Because the principles and working mechanisms of optical sensors have been discussed in many excellent reviews, we focus here on their applications for detecting toxic materials and recent progress in optical chemical sensors [14,28,29,30,31,32].

### 3.1. Fiber Optic Chemical Sensors

Optical fibers are the most widely investigated platform for optical chemical biosensors. Optical fibers are used as sensing elements to measure physical properties such as fluorescence, absorption, and reflectance. Although the different subclasses of fiber-optic chemical sensors have the same working principle, the target specificity and selectivity are determined by the fabrication of optical fibers. Fiber-optic chemical sensors (FOCS) represent an area of active research that has led to several new chemical sensors [14,33,34,35]. The optical fiber technology was coupled with surface plasmon resonance (SPR) to generate different novel fiber-optic sensors. Since the characteristics of SPR were varied by optic structures and metal fabrication on fiber optics, the novel SPR-based, fiber-optic sensors have been developed. Boruah et al. reported a SPR-based, fiber-optic sensor for monitoring lead in water samples [36]. They used U-shaped optic fiber fabrication with chitosan and glutathione as sensing elements and detected ppb ranges of lead ions employing SPR as a transducing element. In this regard, it was speculated that the new chemical sensors would be obtained by coupling new sensing elements and signal transducing elements if the appropriate sensing elements were developed. Additionally, advanced techniques such as microfluidic fabrication, lossy mode resonance, and nanoparticle fabrication have emerged to enhance the sensitivity and selectivity of FOCS [37,38,39]. However, new types of optical chemical sensors continue to emerge that combine various types of sensing and transducing elements. Table 1 lists existing types of optical chemical sensors according to their sensing element and transducer type.

### 3.2. Microfluidic System-Based Optical Chemical Sensors

Among the new chemical sensors, biochip- and microfluidic-based optical chemical sensors can rapidly and conveniently detect and monitor various environmentally toxic materials [52,53]. Since microfluidic devices handle micron-scale samples using microfluidic channels, it has the advantage of requiring fewer samples and having faster analytic processes. Although the structure of fiber-optic microfluidic sensors differs from that of typical FOCS, they can also target toxic materials such as antibiotics, bisphenol A, and various pharmaceuticals with comparable detection limits. Similar to other chemical sensors, microfluidic devices would act as sensing platforms by integrating sensing elements into microfluidic devices. Recently, Li et al. fabricated a microfluidic chip-based, fiber-optic sensor for detecting the antibiotic minocycline [42], which comprised an optical fiber with a microstructured polymer, fabricated using polymethyl methacrylate and polystyrene, to generate an in-fiber optofluidic chemiluminescence device for minocycline detection. A luminol solution was added to report changes in chemiluminescence signals against minocycline concentrations. The sensor exhibited a minocycline detection limit of 100 ppb, which is comparable to that of high-performance liquid chromatography-based analysis [54]. Wang et al. has reported heavy-metal detecting sensors based on a paper-based microfluidic device [43]. They generated heavy metal monitoring microfluidic-based sensors by fabricating metal selective chromogenic reagents on paper-based microfluidic devices. And, it showed 0.29 ppm, 0.33 ppm, and 0.35 ppm detection limits for Cu(II), Ni(II), and Cr(VI), respectively. Consequently, it was emphasized that the fusion of scientific technologies from different research fields makes chemical sensors more advanced. Besides the microfluidic sensors based on the optic fiber mentioned here, many FOCS have been coupled with microfluidic systems for the detection of toxic materials [47,55,56].

FOCS are classified via their target sensing elements, sensing materials, and detection principles [55,56]. As shown in Figure 1, the target sensing element is used to identify chemical compounds, functionalized nanomaterials, aptamers, antibodies, proteins, and even cells. The target sensing elements are coupled with sensing materials such as nanoparticles, luminol, graphene oxide, carbon nanotubes (CNTs), and microfluidic devices, which transduce changes to the signals [57]. Therefore, the targets of chemical sensors are determined by the specificity and selectivity of the sensing elements. As such, it is critical to develop and investigate new sensing elements to expand the range of sensor targets. Once target-specific sensing elements are prepared, they can be coupled with different techniques to generate various types of chemical sensors.

### 3.3. Nanoparticle-Based Optical Chemical Sensors

Optical chemical sensors are based on changes in electromagnetic radiation. Among the many detection principles related to optical properties, colorimetric and fluorometric changes are suitable for the detection of toxic materials. Chemical sensors based on these principles must be equipped with a source for excitation and a detector [58,59]. However, samples must include species that exhibit intrinsic fluorescence. Thus, it is critical to endow samples with fluorescent properties for colorimetric and fluorometric measurements. Gold nanoparticles (AuNPs) are suitable sensor materials because they exhibit size-dependent colorimetric and fluorescent properties [60,61].

He et al. reported that rhodamine B functionalized AuNPs can be used in microfluidic devices to detect Hg ions [46]. Because AuNPs show different colorimetric changes according to their functionalization, size variation, and dispersion rate, AuNP-based chemical sensors have been developed to detect various targets, such as ribonucleotides, ascorbic acid, Cr, Pb, and glucose [62]. Although AuNPs are employed as signal-producing elements in chemical sensors, the target selectivity is determined using their functionalization/fabrication. For example, cysteine-capped AuNPs have been used for ethyl parathion and malathion detection [63,64], and DNA hybridization on AuNPs has been used for Hg ion detection [48]. Consequently, new functionalization (fabrication) technologies for AuNPs have accelerated the development of AuNP-based chemical sensors and expanded the range of targets, not only heavy metal ions but also various other chemicals [65,66,67]. The type of transducer used in these chemical sensors is related to the target sensitivity. For example, the sensitivity of AuNP-based chemical sensors toward Hg ions was enhanced from 5 μM to 0.002 μM of the detection limit by employing a surface-enhanced Raman as the transducer [68]. Developing chemical sensor based on metal nanoparticles including AuNPs and AgNPs is a state-of-the-art multidisciplinary science; thus, it is not reasonable to classify them just by the type of transducers. Rather, it would be better to put them into nanomaterial-based sensors, discussed in Section 3.4.

### 3.4. Nanomaterial-Based Optical Chemical Sensors

The rapid development of new materials and technologies in different research fields has diversified the range of materials and fabrication methods available for sensing elements. Notably, techniques for the fabrication of nanomaterials and nanostructures have opened the door to new sensing elements that can recognize various targets [69,70]. For example, metal nanoparticles, CNTs, metal oxides (quantum dots), and metal organic frameworks are current areas of active research in nanomaterials [70,71,72]. Sensor applications and target selectivity are determined by the type of fabrication and conjugation of nanomaterials. For example, carbon nanomaterials (CNMs) have been used to construct carbon dots, CNTs, graphene, and carbon black, all of which have different properties and are employed in various chemical sensors as sensing elements [73,74]. Carbon dots have been used to develop sensors for detecting cations and anions as well as small molecules and drugs with surface modifications [75,76]. CNTs have also been used as sensing elements with various modifications to detect gaseous analytes such as NH3, NO2, and CO, as well as biomolecules such as boronic acid and glucose [77,78].

Recently, optical sensors based on nanostructured cage material were developed by Balaji et al. for detecting toxic metal ions [25]. Heavy metal ions are typically determined using atomic absorption and emission spectroscopy. However, methods with greater sensitivity and simplicity are required because traditional methods are expensive and complicated. Nanomaterial-cage-based chemical sensors comprise optical-sensor-based cubic Fm3m cage monoliths with different fabrications as sensing elements that use colorimetric changes for signal transduction. Briefly, cubic Fm3m cage monoliths were designed as platforms, and dithizone, TMPyP (α, β, γ, and δ-tetrakis(1-methylpyridinium-4-yl) porphinep-toluenesulfonate), pyrogallol red, and tetraphenyl porphine tetrasulfonic acid were used as cages for detecting Pb, Cd, Sb, and Hg, respectively. The greatest advantage of the nanomaterial-cage-based sensor is that it enables target detection with a high quantification limit by the naked eye.

In addition to the studies mentioned here, many review papers have focused not only on specific nanomaterial-based chemical sensors but also on their working mechanisms, target selectivity upon fabrication, and applications [69,70,79]. Although some aspects of nanomaterial-based chemical sensors are discussed in this review, it should be noted that all chemical sensors share similarities in their basic principles, and the potential of chemical sensors continues to expand along with technological advances.

## 4. Electrochemical Sensors

Electrochemical sensors are an important subclass of chemical sensors that use electrodes as signal-transducing elements [12,80]. Electrochemical sensors can be further divided into potentiometric sensors, voltametric sensors, and conductimetric sensors. These sensors measure the changes in a potential signal caused by an ion-recognition event, whereby the potential between two electrodes causes the oxidation (or reduction) of an electroactive species, with the resistance representing the signal-transducing element [81,82]. Electrochemical sensors measure changes in electrochemical properties induced by the interactions between sensing elements and targets and are further classified based on the type of sensing element, which includes nanomaterials, biomolecules, CNTs, graphene, and optical chemical sensors. Advances in the fields of materials sciences, electrical engineering, device fabrication, and physical chemistry have continually improved the signal transducers and sensing elements of electrochemical sensors. Table 2 lists the types of transducers, sensing elements, and targets of electrochemical sensors.

As many toxic materials are released from anthropogenic activities, heavy metal(loid)s (such as mercury, lead, cadmium, arsenic), chemicals (such as phenolic compounds, nitroaromatics, organophosphorus),and pesticides are increasingly found in environmental systems. Therefore, electrochemical sensors play a pivotal role in avoiding the impact of toxic materials by enabling constant monitoring via simple and fast analytical tools.

### 4.1. CNM-Based Electrochemical Sensors

Electrochemical sensors are defined as chemical sensors that employ potentiometric, amperometric, and conductometric changes as transduced signals, affording them superior sensitivity to optical chemical sensors. Nonetheless, electrochemical sensors are similar to optical chemical sensors in terms of sensing element advances and diversity. Recent advances in nanomaterial science have provided various materials for sensing elements, including metal nanoparticles (NPs) and CNMs such as carbon dots, CNTs, and graphene [26,93,94]. The integration of fabrication technologies has also enabled the use of nanostructured materials as sensing elements in chemical sensors [95,96].

As for optical chemical sensors, CNMs are widely used as sensing elements in electrochemical sensors [97,98]. Carbon nanostructures used for electrochemical sensors include fullerenes, CNTs, graphene, nanocones, and carbon dots, which are classified in terms of their size and shape. In general, nanostructured carbon is applied to electrodes via a fabrication process to detect targets such as pesticides, toxic chemicals, and heavy metal ions. Recently, Wu et al. reported CNT-based electrochemical sensors for measuring the cytotoxicity of 2,4,6-trichlorophenol, bisphenol AF, and polystyrene nanoplastics [83]. The electrodes were modified with tungsten disulfide nanosheets/hydroxylated multi-walled CNTs (WS2/MWCNTs-OH) for enhanced sensitivity. A recent study reported the development of laser-induced graphene printed on polyimide films for 4-nitrophenol detection in water [84]. The presence of 4-nitrophenol induced a cyclic voltammetry response that corresponded to the concentration of 4-nitrophenol. In addition, several studies and review papers have been written on CNM-based sensors [97,99,100]. Owing to their diverse intrinsic electronic and optical properties, chemical versatility, and stability, CNMs are attracting increasing research attention, with new findings on CNM-based chemical sensors also reported.

### 4.2. NP-Based Electrochemical Sensors

Recently, the use of NPs has increased in a variety of fields, including environmental, pharmaceutical, medical, and material sciences, as well as the cosmetics industry [101,102,103,104]. The wide application of NPs is related to their novel properties, which depend on the shape and size of the particles [105]. Thus, methods of synthesizing NPs represent a key research area [106,107]. Typically, NPs are divided into metal-based, metal-oxide-based, and carbon-based NPs [108]. Because the application of CNMs to electrochemical sensors was addressed in the previous section, only electrochemical sensors employing metal- and metal-oxide-based NPs are discussed in this section.

Among the metal-NP-based electrochemical sensors, AuNPs have received the most attention. Castañeda et al. reported AuNP-based electrochemical sensing of DNA [88], and Zhao et al. reported hydrazine detection using an AuNPs/CNTs-ErGO electrochemically reduced graphene oxide composite film [89]. In both cases, the AuNPs were employed as sensing elements to recognize targets. Silver nanoparticles (AgNPs) have also been used in electrochemical sensors [108]. Similar to AuNPs, AgNPs were fabricated on the electrodes of electrochemical sensors to serve as target sensors. AgNP-based electrochemical sensors targeting DNA, chemicals, and pesticides have been developed with variations in electrode types and other modifications [90,109].

Similar to NPs, metal oxides have been applied in various fields because of their novel characteristics, which are determined by their shape and size [110,111]. Among the metal oxides, the use of zinc oxide (ZnO) for sensing toxic materials has been extensively discussed in review articles [70,112,113,114]. ZnO is inexpensive and can be used to fabricate various nanostructures in a relatively simple manner. The shapes of ZnO nanostructures range from 1-D to 3-D, with each shape exhibiting distinct properties. For example, a thin layer of ZnO on the sensor surface enhances the electrochemical response to Hg by facilitating the migration of electrons between the redox-active analytes [115]. Moreover, Ibrahim et al. synthesized cauliflower-shaped ZnO and used it to modify electrodes for the detection of picric acid [91]. Many other types of ZnO have also been reported and employed for electrode modification. Unlike other nanostructured materials, ZnO plays a role in both target selectivity and transduced-signal amplification.

The versatility of nanomaterials, including metal NPs and metal oxides, has been accepted and proven in different research fields, which has accelerated research into their development and application. Advances in nanomaterials science have also provided significant benefits to many different research fields and industries, with their potential as components of chemical sensors being particularly important. Although metal NPs act to enhance the transducing signals through their integration with electrodes, they can also function as sensing elements with improvements to the chemical modification and surface fabrication of NPs. Although it is difficult to endow NPs with target selectivity, studies have achieved target sensitivity by conjugating NPs with organic ligands as well as biological molecules, such as chitosan, DNA, and proteins [116,117,118]. If NPs were conjugated with biomolecules and employed as sensing elements in chemical sensors, they could be classified as biosensors. In this regard, we discussed the application of biomolecule conjugated NPs further in Section 5 with biosensors.

## 5. Biosensors

**Figure 2:**
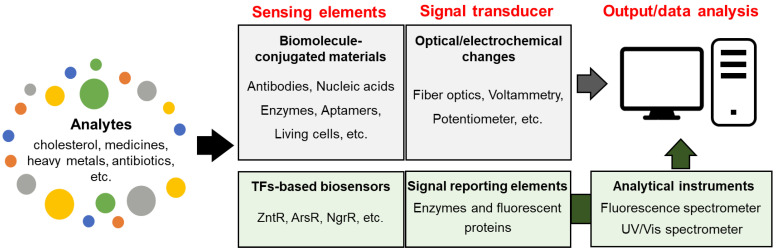
The types of biosensors based on ability to transduce signals into digitized values.

Biosensors targeting biomolecules of interest are usually considered a subclass of chemical sensors because they use the same target sensing and transduction methods [119]. As shown in Figure 2, biosensors are divided into two categories based on their ability to transduce signals into digitized values. One category includes biosensors that integrate sensing and transducing elements with analytical devices, whereas the other requires additional instruments to read the transduced signals. The former type employs biomolecules and biochemical or biological mechanisms as sensing elements and recognition systems and are, therefore, considered to be conventional biosensors [120]. The latter are biosensing systems based on transcription factors (TFs), such as whole-cell biosensors, which require equipment to read the transduced signals. In this regard, various chemical sensors that use biomolecules, such as DNA, enzymes, and antibodies, for target recognition are considered biosensors [121,122,123]. In contrast, TF-based biosensors are not fully accepted as chemical sensors, despite target recognition occurring through the transduction of enzymatic activities and fluorescence. Nonetheless, TF-based biosensors are included in this review because the sensing systems can be chemical sensors equipped with sensing and signal-transducing elements.

### 5.1. Optical Biosensors and Electrochemical Biosensors

As discussed above, optical and electrochemical sensors are classified according to the method of transducing signals indicating the interaction between targets and sensing elements. In general, optical biosensors are based on technology that detects evanescent-wave changes caused by the interaction between targets and sensing elements incorporated with biological molecules [124,125]. Surface plasmon resonance, optical waveguides, optical resonators, and optical fibers have all been used as transducers to measure changes in the reflective index, fluorescence, Raman scattering, and optical absorption upon target recognition [126]. Electrochemical biosensors also employ biomolecules as sensing elements and electrodes to measure electrochemical properties such as voltametric, potentiometric, and electrometric changes as transduced signals [127,128,129]. Although different technologies have been employed for biosensors, the range of targets is determined by the sensing elements coupled with various biomolecules, such as antibodies, enzymes, DNA, aptamers, and cells. As biomolecules are more specific and selective to targets than other materials, biomolecule-coupled chemical sensors are a key area of research. Since the targets of biomolecules are diverse such as chemicals, heavy metals, chemicals, other proteins, and cells, the detecting ranges of targets were expanded. Therefore, the type and integration method of biomolecules used in chemical sensors are critical considerations. Table 3 lists the different types of biosensors according to their sensing and transducing elements.

### 5.2. Enzyme-Based Biosensors

Because enzymes have specific targets, they are attractive for use as sensing elements in chemical sensors. The role of enzymes in chemical sensors is to sense targets and transduce signals based on their activities [14,145], with enzymes such as hydrolases, oxidoreductases, and transferases all used as bioreceptors for sensing targets. Enzyme activities induce optical or electrochemical changes and can be used to detect heavy metals, pharmaceuticals, and phenolics [146].

For example, cholinesterase is widely used as a sensing element to detect toxic materials such as pesticides, heavy metals, and toxins because its activity is inhibited by toxic materials [147]. Recently, Loewenthal et al. used acetylcholinesterase (AChE) coupled with a near-infrared, fluorescent, single-walled carbon nanotube optical sensor. AChE releases thiocholines from acetylthiocholine, increasing near-infrared fluorescence and, thereby, detecting AChE inhibitors by measuring the decrease in signals [130,148]. Another example of an enzyme used in chemical sensors is toluene monooxygenase for toluene detection [131]. This enzyme recognizes and degrades toluene, resulting in the consumption of oxygen, then induces changes in the phosphorescence intensity, which act as transduced signals.

The greatest advantage of enzymes is their target selectivity and enzymatic activity. Enzymes are integrated into chemical sensors to detect targets and produce output signals induced by catalytic activity [149]. However, the stability of enzymes would be an obstacle to enlarging their application because the enzymatic activity is dependent on the stable structure of enzymes. Thus, it would be necessary to consider the stability of enzymes during the integration on the components of sensors. Nonetheless, the advantageous aspects of enzymes accelerate the application of enzyme-based biosensors not only for the detection of toxic materials but also for biomedical analysis [150,151]. As biomolecules such as specific proteins and metabolites indicate certain diseases, enzymes that recognize these molecules have been employed as sensing elements in chemical sensors for biomedical analysis.

### 5.3. Biomolecule-Based Biosensors

Biomolecules such as DNA, peptides, antibodies, and aptamers have been employed as target-sensing elements in chemical sensors [121,152,153]. Target-specific biomolecules are integrated into sensing devices as sensing elements. Changes in the chemical properties induced by the interaction with targets are then transduced by various transducing elements. The basic components are the same as those of other chemical sensors; however, the biomolecules are used for sensing the targets. This type of sensor can also be classed as optical or electrochemical sensors according to the type of signal transducer.

Unlike enzymes, the biomolecules such as DNA, peptides, antibodies, and aptamers possess more versatile natures to modulate target selectivity and sensitivity. In this regard, those biomolecules were actively investigated as sensing elements for chemical sensors. With advances in nanomaterial fabrication technologies, various materials have been conjugated onto nanomaterials. In addition to chemicals and functional groups, biomolecules, such as DNA, antibodies, and aptamers, have been conjugated as ligands onto nanomaterials [123,154]. For example, Liu et al. developed mercury-sensing electrochemical sensors by integrating DNA strands onto cuprous oxide/nanochitosan composites [133]. Interfacial changes on the surface of the electrode caused by DNA–Hg interactions were measured using electrochemical impedance spectroscopy, which showed a nanomolar range of sensitivity. Aptamers, also known as chemical antibodies, are versatile materials used as sensing elements in chemical sensors. Aptamers are single-stranded DNA/RNA oligonucleotides with the advantages of low production costs, versatile applications, and easy modification and operation [155]. Wang et al. and Hu et al. have reported aptamer-based biosensors for monitoring *Pseudomonas aeruginosa* with 10 CFU/mL of detection limit using CDs and biotin integrated aptamers, respectively [135,136]. To facilitate the use of aptamers in chemical sensors, techniques for conjugating aptamers with nanomaterials have rapidly improved. Consequently, aptamer-conjugated NPs, CNMs, and quantum dots have been employed as elements in various chemical sensors [156,157,158]. For example, aptamer-based chemical sensors were applied to detect heavy metals, cancer cells, and various proteins according to the specificity of the aptamers. In addition to the biomolecules mentioned here, many other chemical sensors employ biomolecules as components, such as antibodies, proteins, chitosan, and carbohydrates. By integrating these biomolecules into sensing elements, biosensors can be used to detect a wide range of targets, including toxic gases, heavy metals, phenolic compounds, proteins, and even cancer cells.

Researchers have also reviewed chemical sensors with antibody-, aptamer-, and DNA-based sensors, emphasizing the huge potential of integrating biomolecules with nanomaterials, including CNTs, various NPs, quantum dots, and different types of electrodes for detecting toxic materials [121,159,160].

### 5.4. TF-Based Biosensors

Unlike the chemical sensors discussed above, TF-based biosensors do not transduce signals to digitized values but still comprise sensing and signal transduction elements [161,162]. The sensing elements are TFs, and targets interacting with the TFs are turned on or off via the transcription of genes. The target–TF interaction is indicated by gene expression; therefore, the gene expression level is the signal output [163,164]. For this reason, TF-based biosensors are often called bioreporters and are coupled with additional instruments such as UV/Vis spectroscopy and fluorescence spectroscopy.

Among the TF-based biosensors, whole-cell-based biosensors have been intensively investigated. In these biosensors, the cells are used as sensor platforms to serve as both sensing and signal-transducing elements [165]. Typically, the TFs are target-sensing elements and genes that encode enzymes or fluorescent proteins as signal-transducing elements. As TFs are involved in external stimuli, cells possessing genetically engineered TFs and reporter genes are responsible for specific stimuli. Thus, specific toxic material-sensing biosensors have been developed by employing TFs that respond to toxic materials as sensing elements. TF-based biosensors target a wide variety of materials, including biomolecules and cellular metabolites, but the targets are restricted to toxic materials in this review. The TFs and their corresponding toxic materials are listed in Table 3. For example, *Escherichia coli* contains operons responsive to arsenic and manganese, which are regulated by ArsR and MntR, respectively. When reporter genes such as egfp are inserted under the promoter regions of operons, *E. coli* cells serve as sensors to detect arsenic and manganese based on changes in fluorescent signals [142,166]. In addition, the applications of whole-cell biosensors can be increased by integrating cells with electrochemical devices. Because the cells produce electrochemically active materials upon exposure to toxic materials, the signal changes indicate the presence of toxic materials in samples. However, as the target recognition processes occur inside cells, targets impermeable to cells are not detected by whole-cell-based biosensors. To overcome this disadvantage, cell-free sensing systems that use a mixture of components are required for transcription and translation [167,168]. Alam et al. and colleagues have reported a cell-free, TFs-based sensor system named ROSALIND (RNA output sensors activated by ligand induction) [143]. The same TFs used as sensing elements for whole cell-based biosensors are employed as sensing elements. The interaction between target TFs induces the production of RNA reporting signals by forming a complex with fluorescent chemicals. Thus, it avoids the issue of cell permeability of the target. Nonetheless, the cell free system has disadvantages such as the purification of TFs and preparation of components for transcription and translation.

TF-based biosensors are a subclass of chemical sensors that include both sensing and signal-transducing elements. Therefore, similar to the chemical sensors discussed above, diverse targets and improved applications and performance can be achieved by enhancing the sensing elements and signal-transducing technologies. In addition, the target selectivity and specificity of TF-based biosensors could be modulated via genetic engineering of TFs. Although it is challenging to modulate the target interaction of TFs via genetic engineering, the advance in protein modelling and computational analysis makes the process more accurate and efficient. In this way, the performance of TF-based biosensor can be enhanced, and the targets can also be diversified from existing genetic systems. Because biosensors have many advantages over other chemical sensors, especially in terms of their target specificity and selectivity, efforts should be made to further improve biosensors. Moreover, advances in diverse scientific fields can be used to enhance the performance and application of both chemical sensors and biosensors because both sensors share sensing and signal-transducing elements. Consequently, the rapid development of scientific technologies related to chemical sensors will help protect humans from exposure to toxic materials in the environment.

## 6. Conclusions and Future Prospects

Advances in various industrial fields have increased the production of toxic materials. Although systems for the control and monitoring of toxic materials have been established in many countries, attempts to reduce the threat to human health have been hindered by the rapid increase in toxic materials. It is impossible to identify all toxic materials, but fast and convenient detection methods are vital for effective monitoring. Moreover, technologies must be developed to respond to and monitor newly generated toxic materials. Chemical sensors are commonly used to detect toxic materials and can, therefore, address these concerns. As described in this review, chemical sensors have undergone rapid improvements, and their applications have increased substantially following advances in different scientific fields. The basic structure of all types of chemical sensors is the same, comprising sensing and signal-transducing elements; however, these elements vary widely between sensor types. Although the chemical sensors discussed here were classified just by types of sensing elements and signal transducing elements, it would not be meaningful these days. To enhance the performance of chemical sensors, it is critical to integrate the interdisciplinary sciences as well as to upgrade sensing and signal transducing elements. In addition, the application fields of chemical sensors have been expended rapidly along with the advances in sensing and signal-transducing elements. Recently, various chemical sensors have been applied to medical sciences to diagnose diseases by monitoring biological markers and pathogenic markers [169,170]. Meanwhile, it has been also reported that whole-cell-based biosensors were used to monitor the toxicity and genotoxicity of harmful materials [171,172]. In this way, the chemical sensors could contribute to secure and improve the human health.

Consequently, the future prospects of chemical sensors not only depend on improving sensing elements and integrating them with signal-transducing elements but also integrating advanced technologies. Although this review only discusses a small number of chemical sensors, we focus on the similarity of chemical sensors in terms of their basic principles and future goals. As such, this review provides a unique perspective on chemical sensors and contributes to their continued development.

## Figures and Tables

**Table 1:** List of optical chemical sensors classified by type of sensing element and transducer.

Type of Transducer	Sensing Element	Target	LOD/Detection Ranges	Optical Response	Ref.
LMR-based refractometer	Indium tin oxide NPs Zinc oxide nanorods	Hydrogen gas Sulfide gas	- -	LMR	[40] [41]
SPR based-optic fiber	Graphene film Chitosan-optic fiber	Streptavidin Pb(II)	- 1–7 ppb	Reflective index	[35] [36]
In-fiber optofluidic device	mPOF	Minocycline	100 ppb	Chemilumin.	[42]
Microfluidic device	Chemicals	Cu(II), Ni(II), Cr(VI)	0.29 ppm, 0.33 ppm, 0.35 ppm	Colorimetric	[43]
	Zinc microparticles	Nitrate	19 µM	Colorimetric	[44]
	Berthelot reaction	Ammonia	-	Absorbance	[45]
	AuNPs	Hg(II)	-	Colorimetric	[46]
Microfluidic capillary waveguide	Griess reagents	Nitrite	7 ppb	Colorimetric	[47]
Naked eyes/UV-Vis spec.	Nanostructured cages	Sb(III), Hg(II), Pb(II)	33.7 nM, 6.34 nM, 2.38 nM	Absorbance	[25]
Naked eyes	DNA hybridized AuNPs	Mercury ions (Hg^2+^)	0.5 mM	Colorimetric	[48]
Fluorescence spectroscopy	SWCNTs	DNA sequences	4.0 nM	Fluorescence	[49]
Chemiluminescence analyzer Fluorescence spectroscopy	CDs wsNP-CDs	Phenol Trinitrophenol	0.76 mM 23 µM	Fluorescence Fluorescence	[50] [51]

Note: LMR, lossy mode resonance; SPR, surface plasmon resonance; mPOF, microstructured polymer optic fiber; AuNPs, gold nanoparticles; SWCNTs, single-walled carbon nanotubes; wsNP-CDs, water-soluble nitrogen and phosphorous-doped carbon dots.

**Table 2:** List of electrochemical sensors classified by type of sensing element and transducer.

Type of Transducer	Sensing Element	Target	LOD	Electrochemical Response	Ref.
SPCE	WS2/MWCNTs-OH	2,4,6-trichlorophenol	-	Cyclic voltammetry	[83]
		bisphenol AF PSNP	- -		
Electrochemical analyzer	LIG	4-nitrophenol	95 nM	Cyclic voltammetry	[84]
Metal electrode	Cu_2_O-rGO	NO_2_	50 ppb	Resistance	[85]
	Graphene flake	CO_2_	-	Resistance	[86]
GCE	MWCNTs/CuO-Au	4-aminophenol Acetaminophen	0.105 µM 0.016 µM	Differential pulse voltammetry	[87]
	AuNPs/DNA	DNA	0.78 fmol	Cyclic voltammetry	[88]
	AuNPs/CNTs-ErGO	Hydrazine	0.065 µM		[89]
	AgNPs	Pendimethalin Ethyl parathion	36 nmol/L 40 nmol/L	Square-wave adsorptive Stripping voltammetry	[90]
Metal electrode	cauliflower-shaped ZnO	Picric acid Nitrophenol	0.078 mM -	Current voltage technique	[91]
	TiO_2_-CNTs/Pt	H_2_O_2_	0.016 µM	Cyclic voltammetry	[92]

Note: SPCE, screen-printed carbon electrode; WS2/MWCNTs-OH, tungsten disulfide nanosheets/hydroxylated multi-walled carbon nanotubes; PSNP, polystyrene nanoplastics; LIG, laser-induced graphene; rGO, reduced graphene oxide; GCE, glassy carbon electrode; CNTs-ErGO, carbon nanotube electrochemically reduced graphene oxide composite film.

**Table 3:** List of biosensors classified by type of sensing element and transducer.

Type of Transducer	Sensing Element	Target	LOD	Response	Ref.
Fluorescence spec.	ChE-SWCNT	Pesticides Heavy metals	- -	NIR fluorescence	[130]
Optic fiber	Toluene monooxygenase	Toluene	3 µM	Absorbance	[131]
GCE	Urease-polyaniline	Urea	0.1 mM	Cyclic Voltammetry	[132]
Au electrode	DNA-Cu_2_O@NCs	Hg(II)	0.15 nM		[133]
Potentiostat	DNA-ZnO NPs	Yellow fever virus	0.01 µM	Cyclic voltammetry	[134]
Fluorescence spec. SPR	Aptamer-CDs/GO Aptamer-Biotin	*Pseudomonas aeruginosa*	9 CFU/mL 10 CFU/mL	Fluorescence Reflective index	[135] [136]
TFs-based biosensors	ArsR	As(III), As(V)	10 µg/L	Fluorescence	[137,138]
	CueR	Cu(II)	10 nM		[139]
	ZntR	Pb(II), Hg(II), Cd(II)	-		[139,140]
	TbuT	BTEX	0.24 ± 0.22 µM		[141]
	MntR	Mn(II)	0.01 µM		[142]
	MobR TetR mphR	3-hydroxybenzoate Tetracycline erythromycin	2 mM 1.25 µM 50 µM		[143]
	BenR	benzoate	1 nM		[144]

Note: SWCNT, single-walled carbon nanotubes; Cu_2_O@NCs, cuprous oxide and nanochitosan composites; CDs, carbon dots; GO, graphene oxide; SPR, surface plasmon resonance; BTEX, benzene, toluene, ethylbenzene, and xylene.

## Data Availability

Data are contained within the article.
